# Epidemiology and outcomes of traumatic sternal fractures and associated blunt cardiac injury: a nationwide cohort study in the Netherlands

**DOI:** 10.1007/s00068-026-03222-4

**Published:** 2026-06-01

**Authors:** Dorine S. Klei, Kim E. M. Benders, Luke P. H. Leenen, Karlijn J. P. van Wessem

**Affiliations:** Department of Trauma Surgery, University Medical Centre Utrecht, Suite G04.232, Heidelberglaan 100, Utrecht, 3584 CX The Netherlands

**Keywords:** Traumatic sternal fracture, Nationwide registry, Retrospective cohort study, Blunt cardiac injury, In-hospital outcomes

## Abstract

**Purpose:**

Comprehensive data on epidemiology, trauma mechanisms, associated injuries, and outcomes of traumatic sternal fractures are scarce. This study analysed nationwide data to improve diagnosis and management within the Dutch healthcare system.

**Methods:**

This nationwide retrospective cohort study using the Dutch National Trauma Registry included adult patients admitted with traumatic sternal fractures between 2015 and 2023. Patients with prehospital cardiopulmonary resuscitation or penetrating trauma were excluded. Incidence, patient characteristics, trauma mechanisms, associated injuries, and in-hospital outcomes were analysed. Subgroup analyses evaluated patients with concomitant blunt cardiac injury (BCI).

**Results:**

Of 568,399 adult trauma admissions, 4,765 patients (0.84%) sustained traumatic sternal fractures. Median age was 62 years; 60% were male. Motor vehicle accidents (48%) and falls (28%) were the leading mechanisms. 35% were severely injured (ISS *≥* 16). Associated injuries included rib fractures (51%), spinal fractures (36%), and lung contusions (18%). Critical care unit admission was 40%, with median mechanical ventilation duration of 4 days; median hospital stay was 5 days. In-hospital mortality was 5.7%, and 30-day mortality 6.0%. BCI occurred in 9.5% of patients and was associated with a higher number of injuries and increased injury severity, emergency interventions, and critical care admission, but not higher mortality.

**Conclusion:**

Traumatic sternal fractures are uncommon, but the incidence in The Netherlands is gradually rising. Sternal fractures frequently occur with severe multisystem injuries. Patients with BCI showed greater injury severity and resource needs. Future research should focus on criteria and clinical significance of BCI, and sternal fracture-specific outcomes and treatment strategies in large patient cohorts.

**Supplementary Information:**

The online version contains supplementary material available at 10.1007/s00068-026-03222-4.

## Introduction

To effectively identify, diagnose, and manage traumatic injuries, a comprehensive understanding of incidence, patient characteristics, trauma mechanisms, and associated injuries is essential. However, for traumatic sternal fractures in The Netherlands, these data are lacking.

Findings from international studies vary considerably per country [1] and cannot be directly extrapolated to the Dutch healthcare setting, due to differences in logistics and the relatively high population and hospital density. In general, sternal fractures are reported in < 0.5% of trauma patients [2, 3] and 3–8% of blunt trauma patients [4, 5]. However, injury mechanisms [6–9] as well as associated injuries and mortality [6, 7] vary per country, indicating that epidemiology and morbidity of sternal fractures are influenced by societal factors such as socio-economic status, transportation methods, traffic regulations, and healthcare systems [1].

A single-centre study at our level-1 trauma centre in The Netherlands [10] showed that sternal fractures occurred in 2% of trauma patients, with motor vehicle accidents accounting for 75% and falls for 21% of cases [10]. However, even nationally, patient populations differ between trauma centres and regions. A British study demonstrated that the management of sternal fractures showed a large nationwide variation [11].

Morbidity and mortality are usually not caused by the sternal fractures themselves, but by the associated thoracic injuries, such as spinal fractures, rib fractures, and intrathoracic injuries [6, 7, 9, 12]. For instance, associated blunt cardiac injury (BCI) might result in severe cardiac complications, and 24-hour telemetry is currently recommended for suspected BCI, including myocardial contusion [13]. However, the limited understanding of the epidemiology and clinical outcomes of sternal fractures in the Dutch population may contribute to variation in management and outcomes across the country.

The present study aims to address these knowledge gaps by analysing the epidemiology, trauma mechanisms, injury patterns, and in-hospital outcomes of patients suffering from sternal fractures in The Netherlands using data from the prospective Dutch National Trauma Registry (DNTR) [14]. By analysing nationwide data, this study seeks to improve diagnosis and management of traumatic sternal fractures within the Dutch healthcare system.

## Methods

### Study design

A nationwide cohort study was conducted using data from the Dutch National Trauma Registry (DNTR) [14]. The DNTR is a mandatory prospective national quality registry that includes all incidents involving patients with *≥* 1 injury coded by the Abbreviated Injury Scale (AIS 2008) [15], who are presented to any Dutch emergency department (ED) within 48 h of trauma, and are either admitted to the hospital, transferred to another hospital for admission, or succumb to their injuries in the ED.

The DNTR contains anonymised incident-level data rather than patient-level data. Consequently, it is not possible to ascertain whether individual patients are represented more than once. However, for clarity and readability, incident counts are referred to as “patients” throughout this manuscript.

In the present study, all incidents involving adult patients (aged *≥* 18 years at the time of trauma) admitted to a Dutch hospital between 2015 and 2023 with a diagnosis of traumatic sternal fracture (based on the AIS 2008) were included. Patients were excluded if cardiopulmonary resuscitation (CPR) was performed prior to arrival at the ED, due to the high likelihood that the sternal fracture resulted from CPR rather than the initial trauma. Additionally, patients with penetrating trauma mechanism were excluded.

Under the Dutch Medical Research Involving Human Subjects Act (WMO), ethical approval and informed consent were not required, as the DNTR is a mandatory national quality registry that may be used for anonymised patient research. An independent quality assessment by the coordinating centre (University Medical Centre Utrecht, The Netherlands) ensured compliance with all relevant legislation and regulations, including the Dutch Code of Conduct for Health Research, the EU General Data Protection Regulation (in Dutch: AVG), and the Dutch Law on Medical Treatment Agreements (in Dutch: WGBO) (24U-1760).

This study is reported according to the STROBE Statement for cohort studies [16] (Appendix 1).

### Data collection

Patient characteristics included injury year, age at time of trauma, gender, and comorbidities according to the American Society of Anaesthesiologists (ASA) score [17] before trauma. ED baseline physiology included first systolic blood pressure (SBP), first respiratory rate, and first Glasgow Coma Scale (GCS).

Prehospital outcomes were mode of transport, involvement of the Mobile Medical Team (MMT), and prehospital intubation. The Mobile Medical Team allows for rapid assessment and life-saving interventions in the field by a trained anaesthesiologist or trauma surgeon who can be deployed by helicopter or ambulance. The hospital type was also recorded (level-1 or level-2/3 trauma centre).

Injury patterns included trauma mechanism, injury severity, and associated injuries. Mechanisms of blunt injury were further subdivided into type of traffic accident (involving motorised vehicles / (electric) bicycles / pedestrians / other), assault, low-energy fall, high-energy fall, or other. Injury severity was assessed using the Injury Severity Score (ISS, with ISS *≥* 16 indicating polytrauma), and Revised Trauma Score (RTS, a weighted severity scoring tool (0-7.84) based on Glasgow Coma Scale, systolic blood pressure, and respiratory rate).

Associated injuries were evaluated based on the presence and number of AIS-coded injuries in each body region (any severity). Specifically, the presence of rib fractures, flail chest, clavicular fractures, scapular fractures, lung contusions, haemothorax, (tension) pneumothorax, haemopneumothorax, pneumomediastinum, haemomediastinum, blunt cardiac injury (BCI) including myocardial contusion, spinal injuries, and spinal fracture (including fracture severity) were collected.

To distinguish between myocardial contusion and other types of BCI, myocardial contusions were identified through the AIS codes for minor, major, and unspecified contusions. Other type of BCI was determined via AIS codes for unspecified myocardial injury, myocardial laceration, myocardial avulsion, pericardial injury, or pneumomediastinum with cardiac tamponade. Hemomediastinum and pneumomediastinum without cardiac tamponade were not classified as BCI, but recorded as separate associated injuries.

In-hospital outcomes were the conduction of an emergency intervention (< 24 h after ED presentation), including the type of emergency intervention (damage control thoracotomy or laparotomy / craniotomy / orthopaedic damage control / other), admission to a critical care unit (Intensive Care Unit (ICU) / High Care Unit / Medium Care Unit), including length of stay, and requirement and duration of mechanical ventilation at ICU. Total hospital length of stay was also reported (H-LOS, days), as well as Glasgow Outcome Scale (GOS) at time of discharge, destination after discharge, in-hospital mortality, and 30-day mortality.

### Statistical analysis

Primary outcome was the incidence of traumatic sternal fractures, among the general population and among hospitalised adult blunt trauma patients in The Netherlands.

For the general population, annual incidence was calculated using the yearly number of patients with traumatic sternal fracture and the corresponding Dutch population on 1 January. Overall incidence for the study period was determined by dividing the total number of traumatic sternal fracture patients across all study years by the sum of the yearly population sizes from 1 January 2015 to 1 January 2023. Population data per 1 January each year were obtained from the Dutch Population Register [18]. Population incidence was expressed as a rate per 100,000 inhabitants.

For the blunt trauma patient population, incidence was assessed using the number of traumatic sternal fracture patients and the total number of hospitalised blunt trauma patients in the DNTR, per year and for the entire study period, and was expressed as a percentage.

Secondary outcomes were the in-hospital outcomes of patients with traumatic sternal fracture.

Data were analysed using descriptive and comparative statistics. Categorical variables were expressed as ratio and percentage of the study population. Normality of distribution for continuous variables was assessed for each variable by visual inspection of Q-Q plots, box plots, and histograms of the residuals. All continuous variables showed non-normal distribution and were displayed as median [interquartile range, IQR]. For variables with incomplete data, the number and percentage of missing observations were presented in the tables.

Subgroup analyses were conducted for the subgroup of patients with concomitant blunt cardiac injury (BCI). A statistically significant difference was defined as a two-sided p-value of < 0.05. For several variables, a statistically significant difference was obtained despite equal medians and IQRs between groups; these differences were due to subtle data distributional differences in light of a large sample size.

Statistical analyses were performed using R Studio (version 4.3.0; the R Foundation for Statistical Computing, Vienna, Austria).

## Results

### Patient selection

**Fig. 1:**
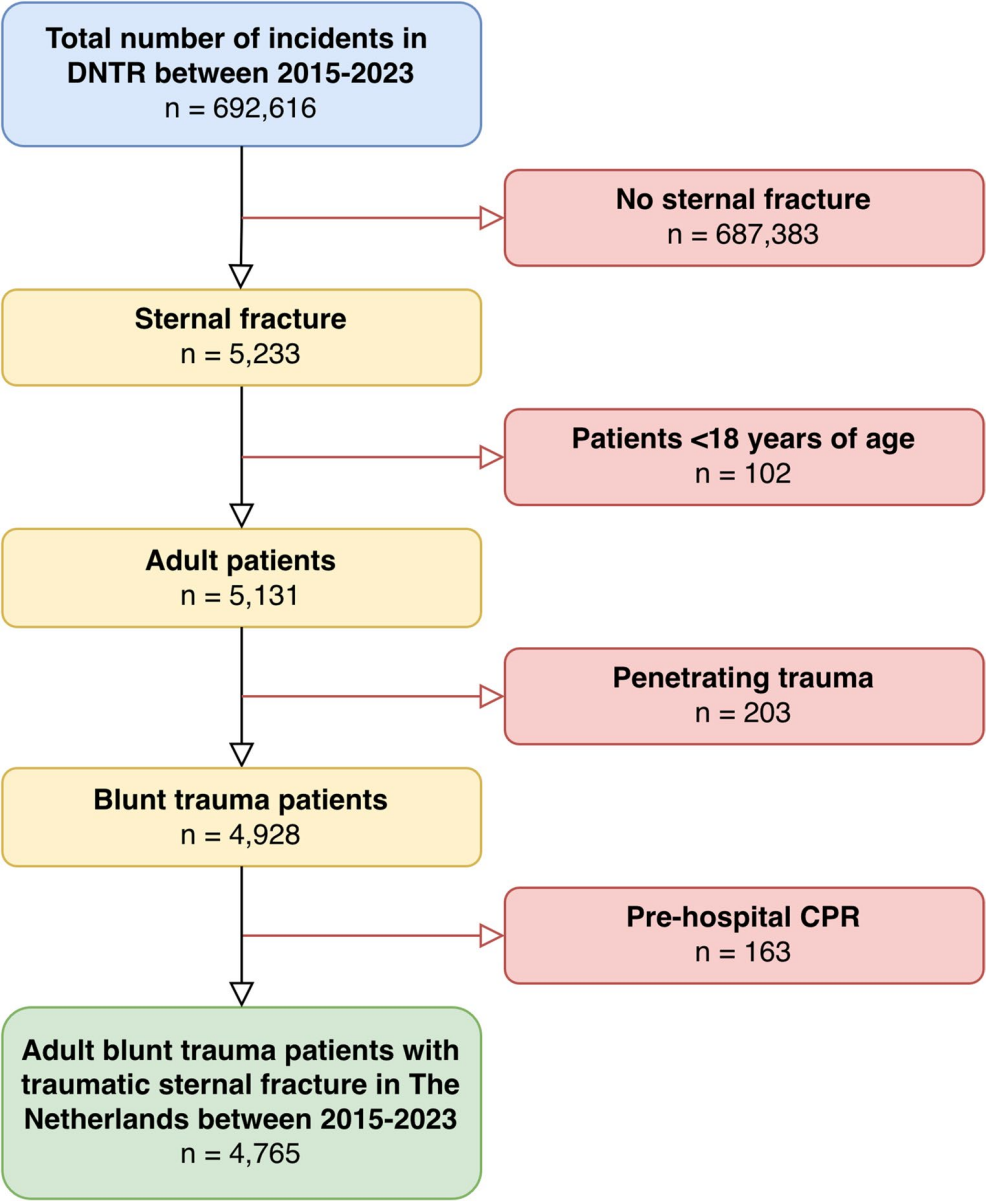
Flowchart of included patients. Abbreviations: DNTR, Dutch National Trauma Registry; CPR, cardiopulmonary resuscitation

During the study period, The Netherlands had 17 to 18 million inhabitants. Between January 2015 and December 2023, a total of 692,616 trauma patients were admitted to all Dutch hospitals. 687,383 patients without sternal fracture were excluded, resulting in 5,233 patients with sternal fracture. Patients below 18 years of age (*n* = 102), patients with penetrating trauma (*n* = 203), and patients treated with CPR before ED-arrival (*n* = 163) were excluded. Finally, 4,765 adult patients with traumatic sternal fracture were included in this study (Fig. 1).

### Incidence of traumatic sternal fracture

**Fig. 2:**
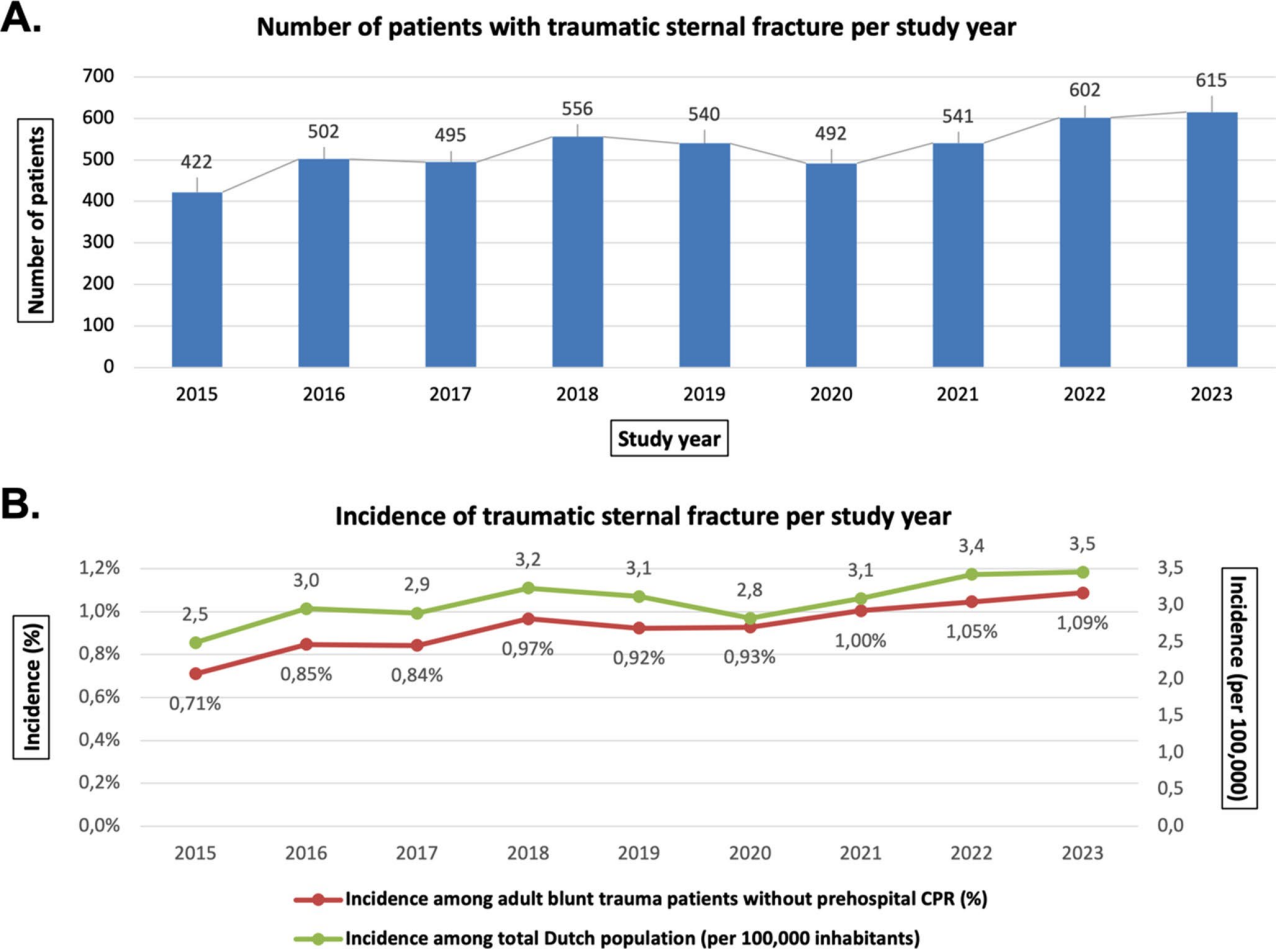
Incidence of traumatic sternal fractures in The Netherlands. (**A**) Number of patients with traumatic sternal fracture per study year. (**B**) Incidence of traumatic sternal fracture among hospitalised blunt trauma patients and the general population per study year. Abbreviations: CPR, cardiopulmonary resuscitation

The total number of traumatic sternal fractures varied annually from 422 in 2015 to 615 in 2023 (Fig. 2A). The incidence increased gradually over the study years. Among hospitalised adult blunt trauma patients, the incidence grew from 0.71% in 2015 to 1.09% in 2023, with a total incidence of 0.93% across the study duration (Fig. 2B). In the general Dutch population, yearly incidence of traumatic sternal fractures increased from 2.5 to 3.5 per 100,000 inhabitants, with an overall occurrence of 3.1 per 100,000 inhabitants across 2015–2023 (Fig. 2B).

### Baseline characteristics and physiology of patients with a sternal fracture

Median age was 62 (45–75) years and 60.1% were male. Comorbidities were mild (ASA 2) in 42.3% of patients, and moderate in 12.8% (ASA 3). Less than 1% had a severe systemic disease (ASA 4) prior to the traumatic event. Since penetrating injuries were excluded, all injuries were caused by a blunt mechanism, with motor vehicle accidents being the most common type of mechanism (48.0%), followed by high energy (14.3%), and low energy falls (13.6%) (Table 1).

The vast majority of patients was transported to hospital by ambulance (85.6%), and the Medical Mobile team (MMT) was involved in 14.1% of transports. Almost 6% of patients were intubated prehospitally (Table 1).

Median first systolic blood pressure was 140 (122–158) mmHg with a median respiratory rate of 18 (15–20) per minute. Median Glasgow Coma Scale (GCS) was 15 (15–15) (Table 1).

**Table 1:** Baseline characteristics and ED physiology

	Total cohort (*n* = 4,765)	No BCI (*n* = 4,312)	BCI (*n* = 453)	*P*-Value
*BASELINE CHARACTERISTICS*				
**Gender (%)**				
Male	2866 (60.1)	2576 (59.7)	290 (64.0)	0.200
Female	1898 (39.8)	1735 (40.2)	163 (36.0)	
Missing or gender neutral	1 (0.0)	1 (0.0)	0 (0.0)	
**Age (years) (median [IQR])**	62 [45, 75]	62 [46, 75]	64 [41, 77]	0.420
**Comorbidity (%)**				
Normal healthy patient (ASA 1)	1708 (35.8)	1539 (35.7)	169 (37.3)	0.076
Mild systemic disease (ASA 2)	2014 (42.3)	1819 (42.2)	195 (43.0)	
Moderate systemic disease (ASA 3)	608 (12.8)	546 (12.7)	62 (13.7)	
Severe systemic disease (ASA 4)	41 (0.9)	35 (0.8)	6 (1.3)	
Moribund (ASA 5)	1 (0.0)	1 (0.0)	0 (0.0)	
Missing	393 (8.2)	372 (8.6)	21 (4.6)	
**Injury mechanism (%)**				
Motor vehicle accident	2286 (48.0)	2023 (46.9)	263 (58.1)	< 0.001*
Motorcycle/Moped accident	240 (5.0)	217 (5.0)	23 (5.1)	
(E-)bike accident	423 (8.9)	400 (9.3)	23 (5.1)	
Pedestrian accident	54 (1.1)	50 (1.2)	4 (0.9)	
High energy fall^a^	681 (14.3)	621 (14.4)	60 (13.2)	
Low energy fall^b^	649 (13.6)	614 (14.2)	35 (7.7)	
Assault	39 (0.8)	33 (0.8)	6 (1.3)	
Other	190 (4.0)	167 (3.9)	23 (5.1)	
Missing	203 (4.3)	187 (4.3)	16 (3.5)	
**Mode of transport (%)**				< 0.001*
Ambulance	4078 (85.6)	3694 (85.7)	384 (84.8)	
Own transport	238 (5.0)	221 (5.1)	17 (3.8)	
Trauma helicopter	57 (1.2)	56 (1.3)	1 (0.2)	
Ambulance assisted by MMT	217 (4.6)	179 (4.2)	38 (8.4)	
Other	10 (0.2)	8 (0.2)	2 (0.4)	
Missing	165 (3.5)	154 (3.6)	11 (2.4)	
**MMT involvement (%)**				
No	3885 (81.5)	3548 (82.3)	337 (74.4)	< 0.001*
Yes	674 (14.1)	573 (13.3)	101 (22.3)	
Missing	206 (4.3)	191 (4.4)	15 (3.3)	
**Prehospital intubation (%)**				
No	3734 (78.4)	3373 (78.2)	361 (79.7)	0.084
Yes	282 (5.9)	248 (5.8)	34 (7.5)	
Missing	749 (15.7)	691 (16.0)	58 (12.8)	
*PHYSIOLOGY IN ED*				
**SBP (mmHg) (median [IQR])**	140 [122, 158]	140 [123, 158]	139 [120, 158]	0.140
Missing (%)	243 (5.1)			
**Respiratory rate (/min) (median [IQR])**	18 [15, 20]	18 [15, 20]	18 [15, 21]	0.004*
Missing (%)	829 (17.4)			
**Respiratory rate (/min) (%)**				
0	7 (0.1)	6 (0.1)	1 (0.2)	0.940
1–5	3 (0.1)	3 (0.1)	0 (0.0)	
6–9	18 (0.4)	16 (0.4)	2 (0.4)	
> 29	121 (2.5)	107 (2.5)	14 (3.1)	
10–29	3789 (79.5)	3429 (79.5)	360 (79.5)	
Missing	827 (17.4)	751 (17.4)	76 (16.8)	
**Glasgow Coma Scale (median [IQR])**	15 [15, 15]	15 [15, 15]	15 [15, 15]	0.108
Missing (%)	462 (9.7)			
**Glasgow Coma Scale (%)**				
3	299 (6.9)	266 (6.8)	33 (7.9)	0.719
4–5	16 (0.4)	15 (0.4)	1 (0.2)	
6–8	47 (1.1)	43 (1.1)	4 (1.0)	
9–12	130 (3.0)	114 (2.9)	16 (3.8)	
13–15	3811 (88.6)	3449 (88.7)	362 (87.0)	
Missing	462 (9.7)	266 (6.8)	33 (7.9)	

Abbreviations: ASA, American society of Anesthesiologists; BCI, blunt cardiac injury; ED, emergency department; MMT, Medical Mobile Team; SBP, first measured systolic blood pressure

*Statistically significant difference (*p* < 0.05)

^a^ High-energy fall: fall from a height of 2–3 times the patient’s body height

^b^ Low-energy fall: fall at the same level

### Injury severity and characteristics

The median Revised Trauma Score was 7.8 (7.8–7.8) and median ISS of the whole cohort was 10 (5–19), with 35% being classified as severely injured (ISS *≥* 16). Median number of AIS-coded injuries was 5 (2–9) per patient. Of all patients with a sternal fracture, 52.0% was admitted to a level-1 trauma centre and 82.4% of all severely injured patients were transported to a level-1 trauma centre (Table 2).

Almost a third of patients suffered from accompanying brain injury (32.5%), 14.7% had abdominal injuries, 35.9% had upper extremity fractures, and 32.2% lower extremity fractures (Table 2).

51% of sternal fracture patients had accompanying rib fractures, and 72% of them had 3 or more rib fractures. 6.5% of patients had flail chest. Overall, 17.6% suffered from lung contusion, of which 40% bilateral lung contusions. Blunt cardiac injury occurred in 9.5% of patients, of whom 95.1% had a myocardial contusion (Table 2).

Other accompanying injuries were clavicular fractures (10.2%), scapular fractures (6.6%), and spinal fractures (36.1%). In total, 22.1% of patients had a spinal body fracture, of which 12.5% were located in the cervical spine, 62.2% in the thoracic spine, and 25.2% in the lumbar spine; 59.9% were classified as major fractures (Table 2).

**Table 2:** Injury characteristics

	Total cohort (*n* = 4,765)	No BCI (*n* = 4,312)	BCI (*n* = 453)	*P*-Value
**RTS (median [IQR])**	7.8 [7.8, 7.8]	7.8 [7.8, 7.8]	7.8 [7.8, 7.8]	0.169
Missing (%)	1296 (27.2)			
**ISS (median [IQR])**	10 [5, 19]	10 [5, 18]	13 [5, 22]	< 0.001*
**Number of AIS coded injuries**				
Total (median [IQR])	5 [2, 9]	5 [2, 8]	7 [3, 12]	< 0.001*
AIS Head (median [IQR])	0 [0, 1]	0 [0, 1]	0 [0, 1]	0.486
AIS Face (median [IQR])	0 [0, 0]	0 [0, 0]	0 [0, 0]	0.063
AIS Neck (median [IQR])	0 [0, 0]	0 [0, 0]	0 [0, 0]	0.007*
AIS Thorax (median [IQR])	2 [1, 3]	2 [1, 3]	3 [2, 5]	< 0.001*
AIS Abdomen (median [IQR])	0 [0, 0]	0 [0, 0]	0 [0, 0]	< 0.001*
AIS Spine (median [IQR])	0 [0, 1]	0 [0, 1]	0 [0, 1]	0.651
AIS Upper Extremity (median [IQR])	0 [0, 1]	0 [0, 1]	0 [0, 1]	0.011*
AIS Lower Extremity (median [IQR])	0 [0, 1]	0 [0, 1]	0 [0, 1]	< 0.001*
AIS External (median [IQR])	0 [0, 0]	0 [0, 0]	0 [0, 0]	0.312
**Severely injured (ISS ≥ 16) (%)**	1670 (35.0)	1475 (34.2)	195 (43.0)	< 0.001*
**Admitted to level-1 trauma centre (%)**	2476 (52.0)	2180 (50.6)	296 (65.3)	< 0.001*
**Polytrauma admitted to level-1 trauma centre (%)**	1376 (82.4)	1204 (81.6)	172 (88.2)	0.023*
*ASSOCIATED INJURIES*				
**Traumatic brain Injury (%)**	1549 (32.5)	1405 (32.6)	144 (31.8)	0.731
**Abdominal Injury (%)**	701 (14.7)	610 (14.1)	91 (20.1)	0.001*
**Upper Extremity Injury (%)**	1712 (35.9)	1527 (35.4)	185 (40.8)	0.022*
**Lower Extremity Injury (%)**	1538 (32.3)	1356 (31.4)	182 (40.2)	< 0.001*
*ASSOCIATED THORACIC INJURIES*				
**Rib Fracture (%)**	2440 (51.2)	2188 (50.7)	252 (55.6)	0.048*
**Rib Fracture ≥ 3 (%)**				0.476
No	530 (21.7)	479 (21.9)	51 (20.2)	
Yes	1760 (72.1)	1571 (71.8)	189 (75.0)	
Unspecified number of rib fractures	150 (6.1)	138 (6.3)	12 (4.8)	
**Flail Chest (%)**	312 (6.5)	278 (6.4)	34 (7.5)	0.386
**Lung Contusion (%)**	839 (17.6)	713 (16.5)	126 (27.8)	< 0.001*
**Type of Lung Contusion (%)**				0.083
Unilateral	409 (48.7)	354 (49.6)	55 (43.7)	
Bilateral	342 (40.8)	280 (39.3)	62 (49.2)	
Unspecified	88 (10.5)	79 (11.1)	9 (7.1)	
**Haemothorax (%)**	142 (3.0)	125 (2.9)	17 (3.8)	0.309
**Major Haemothorax (%)**	14 (9.9)	9 (7.2)	5 (29.4)	0.004*
**Pneumothorax (%)**	954 (20.0)	826 (19.2)	128 (28.3)	< 0.001*
**Major Pneumothorax (%)**	120 (12.6)	107 (13.0)	13 (10.2)	0.374
**Tension Pneumothorax (%**)	51 (5.3)	48 (5.8)	3 (2.3)	0.105
**Haemopneumothorax (%)**	174 (3.7)	150 (3.5)	24 (5.3)	0.050
**Major Haemopneumothorax (%)**	18 (10.3)	12 (8.0)	6 (25.0)	0.011*
**Pneumomediastinum (%**)	103 (2.2)	81 (1.9)	22 (4.9)	< 0.001*
**Haemomediastinum (%)**	47 (1.0)	38 (0.9)	9 (2.0)	0.024*
**Blunt cardiac injury (%)**	453 (9.5)	–	453 (100.0)	NA
**Myocardial Contusion (%)**	431 (95.1)	–	431 (95.1)	NA
**Clavicular fracture (%)**	486 (10.2)	442 (10.3)	44 (9.7)	0.719
**Scapular fracture (%)**	313 (6.6)	292 (6.8)	21 (4.6)	0.081
**Spinal fracture (%)**	1721 (36.1)	1562 (36.2)	159 (35.1)	0.635
**Spinal fracture level (%)**				0.026*
Cervical	664 (38.6)	597 (38.2)	67 (42.1)	
Thoracic	703 (40.8)	653 (41.8)	50 (31.4)	
Lumbar	354 (20.6)	312 (20.0)	42 (26.4)	
**Spinal body fracture (%)**	1054 (22.1)	961 (22.3)	93 (20.5)	0.391
**Spinal body fracture level (%)**				0.041*
Cervical	132 (12.5)	115 (12.0)	17 (18.3)	
Thoracic	656 (62.2)	609 (63.4)	47 (50.5)	
Lumbar	266 (25.2)	237 (24.7)	29 (31.2)	
**Spinal body fracture severity (%)**				0.881
Minor fracture^a^	423 (40.1)	385 (40.1)	38 (40.9)	
Major fracture^b^	631 (59.9)	576 (59.9)	55 (59.1)	

Abbreviations: AIS, Abbreviated Injury Scale; BCI, blunt cardiac injury; ISS, Injury Severity Score; NA, not applicable; RTS, Revised Trauma Score. *Statistically significant difference (*p* < 0.05)

^a^ Minor vertebral body fracture: minor compression (*≤* 20% loss of anterior height)

^b^ Major vertebral body fracture: major compression (> 20% loss of anterior height)

### In-hospital outcomes

Almost 10% of patients underwent an emergency intervention. A total of 39.6% of patients was admitted to a critical care unit; in the entire study cohort, median critical care unit stay was 0 (0–2) days. Of the patients admitted to ICU, 40.4% was mechanically ventilated for 4 (2–11) days. Median length of hospital stay was 5 (2–11) days for the total cohort (Table 3).

The vast majority of patients (64.1%) was discharged to their private home, followed by discharge to a rehabilitation centre (10.9%) (Table 3).

In total, 22.6% of patients made a good recovery, whereas 48.4% of patients had a moderate disability at discharge, 11% was severely disabled, and 0.4% remained in a persistent vegetative state. In-hospital mortality was 5.7%, and 30-day mortality was 6.0% (Table 3).

**Table 3:** In-hospital outcomes

	Total cohort (*n* = 4,765)	No BCI (*n* = 4,312)	BCI (*n* = 453)	*P*-Value
**Emergency intervention (%)**				
None	4057 (85.1)	3689 (85.6)	368 (81.2)	0.002*
Damage control thoracotomy/laparotomy	73 (1.5)	61 (1.4)	12 (2.6)	
Craniotomy	22 (0.5)	21 (0.5)	1 (0.2)	
Damage control orthopaedics	82 (1.7)	76 (1.8)	6 (1.3)	
Other	226 (4.7)	189 (4.4)	37 (8.2)	
Missing	305 (6.4)	276 (6.4)	29 (6.4)	
**Admission to critical care unit (%)**				
No	2722 (57.1)	2530 (58.7)	192 (42.4)	< 0.001*
Yes	1886 (39.6)	1633 (37.9)	253 (55.8)	
Missing	157 (3.3)	149 (3.5)	8 (1.8)	
**Critical care unit LOS (median [IQR])**	0 [0, 2]	0 [0, 2]	1 [0, 4]	< 0.001*
Missing (%)	167 (3.5)			
**Mechanical Ventilation (% of ICU patients)**				
No	696 (48.2)	605 (48.1)	91 (48.9)	0.434
Yes	584 (40.4)	505 (40.1)	79 (42.5)	
Missing	164 (11.4)	148 (11.8)	16 (8.6)	
**Duration of mechanical ventilation (median [IQR])**	4 [2, 11]	4 [2, 11]	5 [2, 10]	0.523
**Hospital LOS (median [IQR])**	5 [2, 11]	4 [2, 11]	6 [2, 14]	< 0.001*
Missing (%)	52 (1.1)			
**Discharge destination (%)**				
Home	3053 (64.1)	2782 (64.5)	271 (59.8)	0.058
Assisted living home	56 (1.2)	53 (1.2)	3 (0.7)	
Nursing home	267 (5.6)	231 (5.4)	36 (7.9)	
Rehabilitation centre	519 (10.9)	457 (10.6)	62 (13.7)	
Other hospital	443 (9.3)	403 (9.3)	40 (8.8)	
Other	103 (2.2)	93 (2.2)	10 (2.2)	
Missing or in-hospital mortality	324 (6.8)	293 (6.8)	31 (6.9)	
**GOS at discharge (%)**				
Persistent vegetative state	20 (0.4)	19 (0.4)	1 (0.2)	0.084
Severe disability	524 (11.0)	459 (10.6)	65 (14.3)	
Moderate disability	2308 (48.4)	2089 (48.4)	219 (48.3)	
Good recovery	1075 (22.6)	974 (22.6)	101 (22.3)	
Missing or in-hospital mortality	838 (17.6)	771 (17.9)	67 (14.8)	
**In-hospital mortality (%)**				
No	4492 (94.3)	4066 (94.3)	426 (94.0)	0.870
Yes	271 (5.7)	244 (5.7)	27 (6.0)	
Missing	2 (0.0)	2 (0.0)	0 (0.0)	
**30-day mortality (%)**				
No	3780 (79.3)	3420 (79.3)	360 (79.5)	0.529
Yes	288 (6.0)	260 (6.0)	28 (6.2)	
Mortality > 30 days	45 (0.9)	38 (0.9)	7 (1.5)	
Missing	652 (13.7)	594 (13.8)	58 (12.8)	

Abbreviations: BCI, blunt cardiac injury; GOS, Glasgow Outcome Scale; ICU, Intensive Care Unit; LOS, length of stay

*Statistically significant difference (*p* < 0.05)

### Subgroup analysis of concomitant BCI

Of all sternal fracture patients, 9.5% (*n* = 453) of patients suffered from blunt cardiac injury (BCI). These BCI patients were more often involved in a motor vehicle accident than non-BCI patients. The MMT was more often involved in transporting these patients to the hospital (22.3% vs. 13.3%, *p* < 0.001). There was no clinically relevant difference in physiology on arrival in ED between BCI patients and patients without BCI (Table 1).

ISS was significantly higher (13 vs. 10, *p* < 0.001) in BCI patients, with a higher number of AIS-coded injuries per patient. BCI patients were also more often severely injured (43.0% vs. 34.2%, *p* < 0.001), and more often transported to a level-1 trauma centre (65.3% vs. 50.6%, *p* < 0.001). They suffered more frequently from associated abdominal injury (20.1% vs. 14.1%, *p* = 0.001), upper extremity injury (40.8% vs. 35.4%, *p* = 0.022), and lower extremity injury (40.2% vs. 31.4%, *p* < 0.001). Furthermore, BCI patients showed a higher frequency of rib fractures (55.6% vs. 50.7%, *p* = 0.048), lung contusions (27.8% vs. 16.5%, *p* < 0.001), major haemothorax (29.4% vs. 7.2%, *p* = 0.004), pneumothorax (28.3% vs. 19.2%, *p* < 0.001), major haemopneumothorax (25.0% vs. 8.0%, *p* = 0.011), pneumomediastinum (4.9% vs. 1.9%, *p* < 0.001), and haemomediastinum (2.0% vs. 0.9%, *p* = 0.024). Moreover, in BCI patients, spinal (body) fractures were more frequently located in the cervical or lumbar spine (Table 2).

BCI patients more often underwent an emergency intervention (12.3% vs. 8.1%, *p* = 0.002), were more often admitted to a critical care unit (55.8% vs. 37.9%, *p* < 0.001), and stayed longer in critical care (1 vs. 0 days, *p* < 0.001) and in hospital (6 vs. 4 days, *p* < 0.001). There was no difference in Glasgow Outcome Scale, nor in mortality (Table 3).

## Discussion

This nationwide cohort study is the first to give a comprehensive overview of traumatic sternal fractures in The Netherlands, including epidemiological features, injury patterns, and clinical outcomes. These data indicate that sternal fractures are uncommon injuries, with an incidence of 0.71%–1.09% among blunt trauma patients and 2.5–3.5 per 100,000 Dutch inhabitants per year, and that this rate is gradually increasing. Patients suffering from sternal fractures were predominantly older adults (median age 62), with a majority of male patients (60.1%). Motor vehicle accidents (48.0%) were the primary trauma mechanism leading to sternal fractures. Significant associated injuries, particularly to the thorax, led to 35.0% of patients being severely injured with an ISS *≥* 16. In total, 39.6% of all patients with a sternal fracture required admission to a critical care unit. Mortality reached up to 6.0% in the first 30 days post-injury, and the majority of patients showed persisting moderate or severe disability at hospital discharge.

In literature, the incidence of traumatic sternal fractures is usually reported as 3–8% among blunt trauma patients [4, 5, 7, 19–23] and < 0.5% among all trauma patients [2, 3, 19, 24]. However, reported rates vary considerably between hospitals, regions, nations, and patient populations. In this inpatient study, incidence was 0.93% among blunt trauma patients. Other reported incidences in single-centre studies vary between 1.4% [25] and 2% [26] for blunt trauma patients, and between 0.0002 [27] and 2.1% [3] for all trauma patients. To date, nationwide epidemiological studies on sternal fractures did not report incidence rates [6, 7, 28, 29].

The variation found between countries can partially be attributed to economic status as described previously in The Global Burden of Disease Study 2019 [1], which reported a decline in sternal and rib fractures in high-income countries and a rise in low- and middle-income countries. The data from the Dutch National Trauma Registry has to be considered in the light of a high-income health care system, and relatively strict traffic regulations (speed checks, mandatory seatbelts, airbags).

The gradually rising incidence of sternal fractures shown in this study corresponds to several other reports in literature. A nationwide German study showed a 15% increase in sternal fractures between 2009 and 2019 [30]. Moreover, universal use of modern multidetector CT-scan (MDCT) reveals more subtle fractures [26]. As a consequence, more sternal fractures may be diagnosed, but might be of limited clinical significance [26].

Motor vehicle accidents were the predominant mechanism of injury in this cohort (48.0%), similar to single-centre studies in the UK [9] and Switzerland [31], but markedly lower than nationwide rates reported in Israel (86.8%) [6] and the US (68.4–84%) [7, 28, 29] and many single-centre studies worldwide [20, 22, 25, 27, 32–37].

Similar to incidence rates, the trauma mechanism of rib and sternal fractures varies significantly between regions worldwide [1], again underscoring that socio-economic status and stricter traffic regulations may have influenced the lower Dutch incidence of sternal fractures.

In our study, the Dutch population that suffered from sternal fractures could be considered a relatively ‘old’ population with a median age of 62, compared to other nationwide studies in Israel [6] (mean 44–48 years) and the USA [7] (mean 51 years). Part of this difference might be attributable to the use of mean instead of median age. However, this high median age contradicts the predominant typical, relatively young male patient [6, 7, 26, 34, 38] suffering from sternal fractures as reported in literature. The higher median age could be due to societal differences specific for The Netherlands, such as higher traffic exposure with increasing age (cycling) and lower occupational hazards, but it is most likely affected by population demographics. A substantial portion of sternal fractures was caused by low energy falls (13.6%) which generally occur more often in the older population due to balance and gait problems.

These findings are exemplary of other Western societies. Mean age in earlier single-centre studies was 45–50 years [27, 34, 38], but over 60 years in more recent studies [3, 9]. A national German study reported 42.3% of sternal fractures in patients over 65 years and highest incidence between 71 and 80 years [39]. In a US multicentre study [3], the majority of patients were over 65 years and experienced worse clinical outcomes despite similar ISS scores.

The 60% male patients in our study are slightly lower than nationwide US studies [7, 28], but similar to an Israeli nationwide study [6]. However, gender dynamics might be shifting, since female patients are reported to have a higher risk of sternal fracture, especially at older age [25, 27, 38].

Consistent with previous studies [6, 7, 9, 26, 28, 29, 32, 37], there was a high rate of associated injuries and over one third of the patients was severely injured. This data reinforces the need for thorough imaging procedures and multidisciplinary assessment in case of sternal fractures.

Blunt cardiac injury (BCI) was found in 9.5% of all patients suffering from a sternal fracture, of which the far majority (95%) consisted of myocardial contusion. This percentage is high in comparison to most nationwide [6, 7, 28] and single centre studies [27, 34, 35, 37]. Only one study reported a comparable incidence, with 8% myocardial contusion and 2.4% cardiac laceration [19]. It remains challenging to compare this number to other cohorts due to the lack of a clear definition of BCI and myocardial contusion [40–42]. As a result, there may have been variations in diagnosis of BCI and myocardial contusion between the different centres included in the DNTR. As the DNTR only contains AIS-coded injuries, the diagnostic criteria underlying these diagnoses are unknown.

At present, the predominant practice is to admit all patients with suspected BCI and myocardial contusion for clinical rhythm observation [13, 40, 41, 43], which may have led to a relative overrepresentation of BCI diagnoses in this nationwide inpatient cohort.

In one nationwide US study [7], mortality was significantly higher in patients with BCI, but the definition of BCI was not specified. In the present cohort, patients suffering from BCI were more severely injured and required more emergency interventions and critical care unit admissions. However, some effect sizes were small. Furthermore, as detailed multivariable analyses were beyond the scope of this descriptive nationwide study, it cannot be established that these differences are directly attributable to BCI or rather reflect the overall higher injury severity in this group.

With spinal fractures in 36.1% and spinal body fractures in 22.1% of patients, the occurrence of concomitant vertebral fractures was higher than in other nationwide studies [6, 28] and single-/multicentre studies [19, 22, 26, 27, 34, 35, 38, 44], although none of these studies specified whether spinal fractures referred to vertebral body fractures or the entire vertebra. Two single-centre studies reported higher rates of spinal fractures: one in the US (38.2%) [3] and one in our own level-1 trauma centre in The Netherlands (45%) [45].

The main strength of this study is its nationwide design, using the mandatory Dutch National Trauma Registry that encompasses all hospital-admitted trauma patients in The Netherlands. This has led to a large representative dataset that allowed for reliable estimation of incidence and outcomes throughout the entire country. Especially for rare injuries such as sternal fractures, studies with large patient populations, are essential to obtain comprehensive and meaningful results. Only very few [6, 7, 28, 29] other nationwide epidemiological studies on sternal fractures have been published, mostly based on national databases without complete population coverage [7, 28, 29].

Nonetheless, this study has several limitations inherent to its retrospective design and its use of a nationwide database with predefined variables. Firstly, the DNTR only included patients that were admitted to hospital. Consequently, patients discharged directly from the emergency department were not included, and the present findings cannot be extrapolated to all sternal fracture patients. Also, no data was available involving readmissions after initial discharge. Secondly, no sternal fracture characteristics, including morphology, location, and displacement, nor fracture-specific treatment and outcomes were available, limiting an in-depth analysis of sternal fracture severity and its association with treatment strategies and outcomes. Thirdly, myocardial contusion and BCI were identified solely from AIS codes, with unknown underlying diagnostic criteria, which may have introduced heterogeneity between centres. Fourthly, several statistically significant differences between patients with and without BCI may have limited clinical relevance due to the large sample size and small absolute effect sizes. Moreover, it is unknown whether any less favourable outcomes in this group are independently associated with BCI or reflect a generally higher injury severity. Nonetheless, the present findings provide insight into injury patterns among patients with BCI and myocardial contusion, which warrants further research. Finally, no data was available on the cause of death in case of in-hospital or 30-day mortality.

Future studies within the field of traumatic sternal fractures should focus on prospective data collection to further investigate the influence of fracture-specific characteristics, pain management, and different treatment modalities. Studies should aim for large, comprehensive cohorts that include all sternal fracture patients, including patients discharged home from ED. More specifically, defining standard diagnostic criteria and protocols for blunt cardiac injury and myocardial contusion is warranted, and will further aid in investigating the clinical significance of these injuries as well as safe discharge criteria for patients with sternal fractures, to ultimately reduce length of hospital stay and unnecessary health care utilisation.

In conclusion, traumatic sternal fractures are relatively rare within the Dutch (trauma patient) population, but incidence is gradually increasing. This first nationwide Dutch study showed that sternal fractures are frequently associated with severe concomitant injuries that may warrant emergency interventions and critical care unit admission. Blunt cardiac injury was present in 9.5% of patients, although its independent impact on clinical outcomes remains uncertain and requires further research.

The incidence and trauma mechanism of traumatic sternal fractures differ significantly between different countries, highlighting the importance of nationwide data that may lead to further improvement of risk stratification, standardising care, and optimising outcomes.

## Electronic Supplementary Material

Below is the link to the electronic supplementary material.

Supplementary Material 1

## Data Availability

The DNTR dataset is not publicly available. Researchers wishing to analyse DNTR data can submit a formal request via https://www.lnaz.nl/trauma/landelijke-traumaregistratie and will receive the results of their requested data analyses. The R script used to generate the results of this study is available from the study authors upon reasonable request.
